# Novel nitrite reductase domain structure suggests a chimeric denitrification repertoire in the phylum Chloroflexi

**DOI:** 10.1002/mbo3.1258

**Published:** 2022-01-13

**Authors:** Sarah L. Schwartz, Lily Momper, Luiz Thiberio Rangel, Cara Magnabosco, Jan P. Amend, Gregory P. Fournier

**Affiliations:** ^1^ Microbiology Graduate Program Massachusetts Institute of Technology Cambridge Massachusetts USA; ^2^ Department of Earth, Atmospheric, and Planetary Sciences Massachusetts Institute of Technology Cambridge Massachusetts USA; ^3^ Exponent Inc. Pasadena California USA; ^4^ Department of Earth Sciences ETH Zurich Zurich Switzerland; ^5^ Department of Earth Sciences University of Southern California Los Angeles California USA; ^6^ Department of Biological Sciences University of Southern California Los Angeles California USA

**Keywords:** Chloroflexi, cytochrome, denitrification, nitric‐oxide reductase, nitrite reductase, phylogeny

## Abstract

Denitrification plays a central role in the global nitrogen cycle, reducing and removing nitrogen from marine and terrestrial ecosystems. The flux of nitrogen species through this pathway has a widespread impact, affecting ecological carrying capacity, agriculture, and climate. Nitrite reductase (Nir) and nitric oxide reductase (NOR) are the two central enzymes in this pathway. Here we present a previously unreported Nir domain architecture in members of phylum Chloroflexi. Phylogenetic analyses of protein domains within Nir indicate that an ancestral horizontal transfer and fusion event produced this chimeric domain architecture. We also identify an expanded genomic diversity of a rarely reported NOR subtype, eNOR. Together, these results suggest a greater diversity of denitrification enzyme arrangements exist than have been previously reported.

## Graphical abstract

Nitrite reductase (Nir) and nitric oxide reductase (NOR) are the two central enzymes in denitrification, a key process in the global nitrogen cycle. This study identifies a novel Nir domain architecture and expanded diversity in a rarely reported nitric oxide reductase variant (eNOR) in members of the bacterial phylum Chloroflexi.

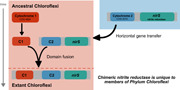

## 1 INTRODUCTION

**Figure 1:**
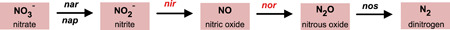
Denitrification. Complete denitrification transforms nitrate into dinitrogen gas. Respiratory nitrate reductase gene (*nar*) and periplasmic nitrate reductase (*nap*) genes are distributed in non‐denitrifying organisms. Nitrite reductase (Nir) is considered the canonical first enzyme of denitrification (Graf et al., 2014), followed by nitric oxide reductase (NOR) and nitrous oxide reductase (Nos)

Microbial denitrification is a key pathway in global nitrogen cycling and has been studied extensively for its role in fixed nitrogen loss and as a source of potent greenhouse gases (Decleyre et al., 2016; Zumft, 1997). Diverse bacteria are capable of denitrification, often facultatively using nitrate or nitrite as an alternative electron acceptor in oxygen‐limited zones. Several diverse microorganisms have the genomic capacity to perform complete denitrification (Figure 1), reducing nitrate to dinitrogen gas (Canfield et al., 2010; Philippot, 2002).

Denitrification has been widely reported in various taxa (Philippot, 2002; Zumft, 1997), and the utility of the pathway is underscored by the diversity of key constituent enzymes. The canonical denitrification enzyme is dissimilatory nitrite reductase, Nir, which reduces nitrite to nitric oxide (NO). Nir functionality is found in two distinct enzymes—the copper‐based nitrite reductase NirK, and the cytochrome‐type reductase NirS (Braker et al., 2000; Decleyre et al., 2016; Priemé et al., 2002). NirS reduces nitrite via cytochrome *cd1*, a dimer of subunits each containing heme *c* and heme *d1*; in canonical denitrification, as observed in *Pseudomonas aeruginosa*, cytochrome *cd1* catalyzes the oxidation of a colocalized cytochrome c551 to reduce nitrite to NO at the heme *d1* site (Philippot, 2002; Zumft, 1997). The *nirS* gene has been reported as a constituent of a larger gene cluster containing genes such as *nirM* and *nirF*, which encode biosynthetic proteins for the cytochrome *c*551 and heme *d*_*1*_, respectively, and the nitrite transporter *nirC* (Kawasaki et al., 1997; Philippot, 2002).

The next step in the pathway—the reduction of NO to nitrous oxide—is catalyzed by nitric oxide reductases (NORs). Most bacterial NORs are homologous and closely related to one another, and to oxygen reductases in the heme–copper oxygen reductase superfamily (Hemp & Gennis, 2008). The most widely studied NOR enzymes are cytochrome‐type nitric oxide reductases (cNOR) and quinol‐dependent nitric oxide reductases (qNOR) (Graf et al., 2014; Hemp & Gennis, 2008; Hendriks et al., 2000), distinguished by their respective electron donors. Enzymes in the cNOR subfamily have two subunits—one catalytic site and one heme‐containing electron shuttle that accepts electrons from cytochrome *c*—while qNOR family enzymes' single, fused subunit accepts electrons from membrane‐bound quinol groups (Hemp & Gennis, 2008). Rarer, alternative NOR enzymes, including sNOR, gNOR, and eNOR, have been more recently identified and characterized in limited members of the Proteobacteria, Firmicutes, Archaea, and Chloroflexi (Hemp & Gennis, 2008; Hemp et al., 2015; Sievert et al., 2008; Stein et al., 2007). Like cNOR, these enzymes are predicted to have a two‐subunit structure, but the second subunit in these NORs contains a cupredoxin instead of heme *c* fold (Hemp & Gennis, 2008).

Many bacteria contain genes encoding only one or a partial subset of the four denitrification steps. Such organisms may perform partial denitrification, while others may use one of these enzymes for nondenitrifying functions (Graf et al., 2014; Hendriks et al., 2000; Roco et al., 2017; Sanford et al., 2012). In partial denitrifiers, the co‐occurrence of denitrification pathway genes appears to vary across different taxa and environments (Graf et al., 2014). Some of this variation may be constrained by the chemistry of certain intermediates. For example, nitric oxide (NO), the product of NirS and NirK, is highly cytotoxic. Both Nir types are periplasmic, and so cells require a means of effluxing or detoxifying NO before it accumulates to lethal levels. Denitrifiers are thought to immediately reduce NO to nitrous oxide (N_2_O) to avoid injury, using membrane‐bound NOR enzymes (Hendriks et al., 2000). Perhaps, for this reason, it is rare to find genomes that contain *nir* but not *nor*, while organisms showing the inverse—the presence of a *nor* gene but not a *nir* gene—are far more common (Graf et al., 2014; Hendriks et al., 2000). While cNORs are only found in denitrifying microbes, other types of NOR—for example, quinol‐dependent qNOR—are found in nondenitrifiers and can presumably detoxify environmental NO (Hendriks et al., 2000). Beyond NORs, alternative pathways to NO detoxification are possible, including alternative enzymes such as cytochrome *c* oxidase (Blomberg & Ädelroth, 2018) or oxidoreductase (Gardner et al., 2002), flavorubredoxin (Gardner et al., 2002), or flavohemoglobins (Sánchez et al., 2011).

While denitrification has been most widely studied and observed in Proteobacteria, the process has also been identified in other phyla, including Chloroflexi. Chloroflexi are ecologically and physiologically diverse, and often key players in oxygen‐, nutrient‐, and light‐limited environments, including anaerobic sludge and subsurface sediments (Hug et al., 2013; Ward, Hemp et al., 2018). Previous surveys have indicated the presence of diverse nitrite reductases in Chloroflexi; members of order Anaerolineales and classes Chloroflexia and Thermomicrobia may have the capacity for nitrite reduction via the copper‐type NirK (Decleyre et al., 2016; Hug et al., 2013; Wei et al., 2015). However, recent studies indicate that certain Chloroflexi—including members of Anaerolineales—may possess *nirS* instead of *nirK* (Hemp et al., 2015; Ward, McGlynn et al., 2018), and may also harbor a divergent variant of *nor* previously reported in members of Archaea (Hemp & Gennis, 2008; Hemp et al., 2015). These findings suggest that the evolution and/or biochemistry of denitrification may be unusual for this subset of bacteria, and informative for a broader understanding of microbial denitrification metabolisms and their origin.

## 2 EXPERIMENTAL PROCEDURES

### 2.1 Genome sampling and assembly

Collection of all fluid samples and total genomic DNA extractions from those fluids, as well as corresponding physical and geochemical data, have been described previously (Heard et al., 2017; Lau et al., 2014, 2016; Magnabosco et al., 2016; Momper & Jungbluth, 2017; Momper & Kiel Reese, 2017; Osburn et al., 2014). All metagenome‐assembled genomes (MAGs) from North America and Africa were reconstructed according to the methods used in Momper and Jungbluth (2017). MAG identifiers and sources are listed in Table A5. Completeness was calculated using the composite values from five widely accepted core essential gene metrics. Duplicate copies of any of these single‐copy marker genes were interpreted as a measure of contamination (Alneberg et al., 2014; Campbell et al., 2013; Creevey et al., 2011; Dupont et al., 2012; Wu & Scott, 2012). Individual genomes were then submitted for gene calling and annotations through the DOE Joint Genome Institute IMG‐ER (Integrated Microbial Genomes expert review) pipeline (Huntemann et al., 2015; Markowitz et al., 2008). For quality control purposes, the genes flanking every denitrification gene presented in this study were individually searched on the National Center for Biotechnology Information's (NCBI) RefSeq database using the BLASTp algorithm, confirming that top hits for all flanking genes were also to Chloroflexi. This step ensured that the nitrogen transforming genes of interest presented here were not simply on scaffolds that were incorrectly binned into a putative Chloroflexi genome.

### 2.2 Genetic database construction and sequence sampling

Sequences for *nirS* and *eNOR* genes from Sanford Underground Research Facility (SURF) MAG 42 (see Table A5) were used as queries to BLAST (Camacho et al., 2009) three genomic repositories:

1. Genome databases constructed for 21 Chloroflexi genomes assembled from deep‐subsurface MAG data (Jungbluth et al., 2017; Momper & Jungbluth, 2017; Table A5).
2. Genome databases constructed for 86 genomes from recent MAG assembled sludge bioreactor genomes (Parks et al., 2017; Table A6).
3. The full NCBI nonredundant protein database (as of September 25, 2019; Agarwala et al., 2018).

Additionally, putative environmental homologs were evaluated using protein sequence data from SURF MAG 42 to query NCBI's nonredundant environmental metagenomic sequence database (env‐nr, as of June 2020; Agarwala et al., 2018). Hits from all databases were combined and assessed for quality; hits with *E* ≤ 1 × 10^−10^ were included for initial analyses. To capture diversity while limiting imprecision and biased sampling of overrepresented groups (e.g., Proteobacteria), hits were subsampled to the genus level, except for members of the Chloroflexi (to fully capture the taxonomic distribution of the novel gene variant). One additional, divergent multispecies hit was allowed per genus. The genus‐level filter was also removed for C1, where non‐Chloroflexi hits were severely limited (see below). Duplicate sequences (from strains with multiple genome entries or in multiple databases surveyed) were removed. Expanded database hits and filtering data are available as Supporting Information Data Files at https://doi.org/10.6084/m9.figshare.14515554.v2.

### 2.3 Sequence alignment

Putative homologous protein sequences were aligned with MAFFT, using autoparameterization (Nakamura et al., 2018), and visualized in Jalview (A. M. Waterhouse et al., 2009). Alignments were manually curated; partial sequences with substantial missing regions or anomalous insertions in conserved regions of the protein were removed to avoid confounding phylogenetic analyses and evolutionary model selection. Protein sequence alignments were trimmed to the length of individual domains identified by NCBI's Conserved Domains Database (CDD). Each domain was then realigned.

### 2.4 eNOR

A preliminary alignment for the *eNOR* gene showed a poorly conserved region near the C‐terminal end of the open reading frame (ORF); to improve accuracy and avoid misalignment, this region was manually removed, and the remaining sequences were realigned before tree construction. Two sequences (Actinobacteria bacterium RBG_16_68_12, OFW73639.1, and *Thermus* WP_015717644.1) with missing N‐terminal regions and three sequences (Chloroflexi bacterium, RME47896.1; Rhodocyclaceae bacterium UTPRO2, OQY7467.1; and *Rhodothermus profundi*, WP_072715415.1) with missing C‐terminal regions were included in the final alignment; the placement of these sequences is therefore based upon fewer alignment sites than other taxa. All retain key active site residues and show no clear evidence of long‐branch attraction artifacts in the tree.

### 2.5 C1

An initial alignment for the C1 domain showed a poorly conserved N‐terminal region. To improve accuracy, this region was manually removed, and the remaining sequences realigned before tree construction.

### 2.6 NirS

Because the NirS domain had a C‐terminal placement in the ORF across hits, C‐terminal sites extending beyond the identified NirS domain were included in the trimmed alignment.

### 2.7 Rooting and outgroup identification

#### 2.7.1 eNOR

Ingroup *eNOR* subunit I sequences were identified by the presence of a conserved Gln residue in alignment position 323. This site distinguishes eNOR not only from other NORs but also from members of the oxygen reductase superfamily, which have a conserved Tyr in this site that plays a role in cofactor crosslinking (Hemp & Gennis, 2008; Table A4). Outgroup sequences (oxygen reductase superfamily or other divergent NORs) were subsampled to a single taxon representative per major subgroup observed in a preliminary tree. Retained outgroup sequences CCQ74688.1, WP_100277903.1, WP_097280063.1, WP_089728124.1, RLC59399.1, and WP_083704903.1 are annotated as uncharacterized domains. The remaining sequences were realigned before tree construction and manually rooted on the branch leading to the outgroup.

#### 2.7.2 C1

Due to a paucity of initial hits (17 total genera), the genus‐level filter was removed for all phyla to increase the resolution of the domain phylogeny. A preliminary tree (Figure A8) was expanded to identify outgroup sequences by including hits with *E* ≤ 10^−4^. The resulting tree was rooted using minimal ancestor deviation (MAD) rooting (Tria et al., 2017).

#### 2.7.3 C2 and NirS

Sequences were rooted using MAD rooting (Tria et al., 2017).

### 2.8 Tree construction

Maximum‐likelihood trees were constructed using IQ‐Tree (Nguyen et al., 2015), under the optimal model defined by the ModelFinder (‐MFP) command (Kalyaanamoorthy et al., 2017; Table A7). Ultrafast bootstraps and approximate likelihood ratio tests were performed using IQ‐Tree's ultrafast bootstrap and Sh‐aLRT parameters (Hoang et al., 2018; Minh et al., 2013). Full domain and gene sequences, sequence alignments, and raw treefiles are available as Supporting Information Data Files at https://doi.org/10.6084/m9.figshare.14515554.v2.

### 2.9 Gene and enzyme structural analysis

FIND (Murali et al., 2019) was used to identify structural features and conserved denitrification pathway genes in deep subsurface genomes. Putative domains within denitrification gene ORFs were identified and compared across genomes using BLAST and NCBI's CDD (S. Lu et al., 2020; Marchler‐Bauer et al., 2015) and EMBL InterPro (Mitchell et al., 2019). C1 from SURF MAG 42 was classified by CDD as COG4654 (*e* = 6.4 × 10^−4^) and by hmmscan (Potter et al., 2018) as cytochrome *c* superfamily (accession 46626) hit (*e* = 1.6 × 10^−5^). C1 did not show a strong pfam match in hmmscan; the closest match was PF13442.8 (independent *e* = 0.18). C2 from SURF MAG 42 was classified by CDD as COG2010 (*e* = 5.31 × 10^−9^), and included an annotated region classified as pfam 13442 (*e* = 4.06 × 10^−7^); C2 was identified in hmmscan as a cytochrome *c* superfamily (accession 46626) hit (*e* = 1.2 × 10^−17^), with a pfam match to PF13442.8 (independent *e* = 2.8 × 10^−10^). While clearly homologous, the C1 and C2 families appear distantly related and do not appear within the other's data set of closely related sequences (see above). Gene neighborhoods were visualized using Gene Graphics (Harrison et al., 2018), using a 20,000 base pair region. Gene and domains identified in each neighborhood were sourced and cross‐referenced with NCBI's RefSeq and CDD (Marchler‐Bauer et al., 2015; O'Leary et al., 2016). Existing enzyme structures for canonical denitrification genes were downloaded from the RCSB Protein Data Bank (Berman et al., 2000). Anaerolineales‐type enzyme structures were predicted using SWISS‐MODEL (A. Waterhouse et al., 2018). All enzyme structures were visualized and analyzed in PyMOL (The PyMOL Molecular Graphics System, Version 2.0, Schrödinger, LLC). SWISS‐MODEL outputs are available at https://doi.org/10.6084/m9.figshare.14515554.v2.

## 3 RESULTS

To investigate divergent denitrification genes in Chloroflexi, we performed a comprehensive analysis of denitrification homologs in over 100 recently sequenced Chloroflexi MAGs, as well as previously available genomes and metagenomes from the NCBI protein databases. Domains of interest were initially identified in SURF MAG 42, an Anaerolineales bacterium sampled in the SURF, a former gold mine in South Dakota (Momper & Jungbluth, 2017).

### 3.1 Apparent chimeric fusion in Chloroflexi NirS

**Figure 2:**
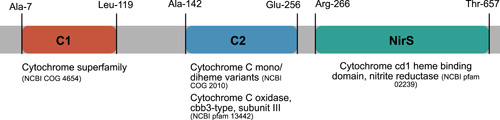
Open reading frame domain map. Conserved domain analysis of SURF MAG 42 Chloroflexi nitrite reductase (GenBank RJP53747.1) indicates the presence of two distinct cytochrome superfamily domains and a C‐terminal nitrite reductase domain. MAG, metagenome‐assembled genome; SURF, Sanford Underground Research Facility

Domain analysis of the Anaerolineales‐type nitrite reductase ORF from SURF MAG 42 indicated three putative functional regions of interest: one cytochrome‐type NirS domain and two cytochrome *c* superfamily domains (Figure 2).

The first cytochrome domain (C1) in the Anaerolineales‐type ORF was identified by NCBI's CDD (S. Lu et al., 2020; Marchler‐Bauer et al., 2015) as a cytochrome c551/552. The second cytochrome domain (C2) was predicted with high specificity as a cytochrome *c* mono‐ and diheme variant. C2 included a region predicted as a cbb3‐type cytochrome *c* oxidase subunit III; such subunits frequently contain two cytochromes (Bertini et al., 2006).

**Figure 3:**
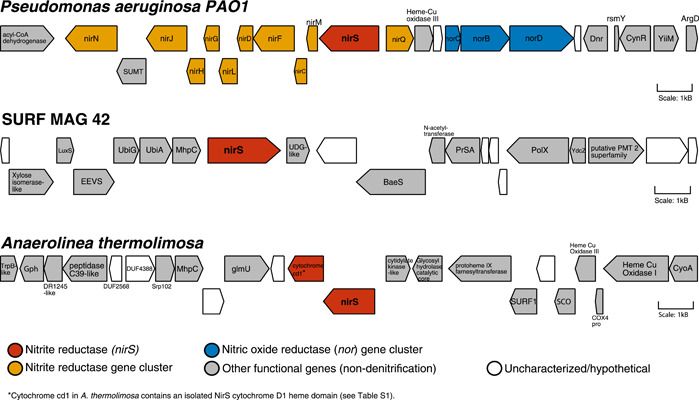
NirS gene neighborhood in SURF MAG 42 versus *Pseudomonas aeruginosa*. Gene neighborhood analyses of the 20,000 base pair region surrounding *nirS* differ markedly between *P. aeruginosa PAO1* (GenBank reference sequence NP_249210.1, top) and two Chloroflexi genomes containing the C1‐C2‐NirS gene: SURF MAG 42 (center) and *Anaerolinea thermolimosa* (bottom). While the *P. aeruginosa* nitrite reductase occurs as part of a larger *nir* operon, and in close proximity to nitric oxide reductase genes, the *nirS* ORF neighborhoods in SURF MAG 42 and *A. thermolimosa* do not appear to contain other denitrification‐specific genes. Detailed descriptions of ORF/gene families and functions can be found in Table A1. MAG, metagenome‐assembled genome; ORF, open reading frame; SURF, Sanford Underground Research Facility

**Figure A1:**
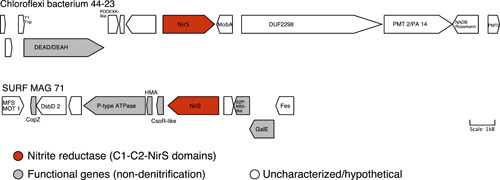
C1‐C2‐NirS gene neighborhoods in Chloroflexi. A gene neighborhood showing the 20,000 base pair region adjacent to the putative nitrite reductase gene containing the C1‐C2‐NirS domain architecture. Neighborhoods are shown for homologs in Chloroflexi bacterium 44‐23 (GenBank accession OJX39483.1) and SURF MAG 71 (GenBank accession RJP52528.1). Expanded descriptions of gene names and functions are provided in Table A2

Gene neighborhood analyses indicated that the MAG‐derived nitrite reductase ORF displays a very different local genomic environment as compared with a canonical nitrite reductase neighborhood in *P. aeruginosa PAO1* (Figure 3). In *P. aeruginosa*, *nirS* (NCBI reference sequence NP_249210.1; O'Leary et al., 2016) co‐occurs with other genes in the *nir* operon and is closely adjacent to genes encoding a cNOR. This arrangement places nitrite reduction in *cis* with NO reduction, the next step of canonical denitrification. In the Chloroflexi MAG, no other denitrification genes appear within a 20,000 base pair neighborhood for the C1‐C2‐NirS nitrite reductase. A similar pattern is observed for a homologous C1‐C2‐NirS nitrite reductase gene identified in the Chloroflexi *Anaerolinea thermolimosa* (Matsuura et al., 2015); the *A. thermolimosa* neighborhood also shows no evidence of other denitrification genes in the immediate vicinity of the novel NirS, though it does contain some ORFs with predicted functionality similar to those in the SURF MAG 42 neighborhood (Figure 3). Full descriptions of all gene abbreviations are provided in Table A1. Analyses of additional selected C1‐C2‐NirS ORF neighborhoods within other Chloroflexi reveal diverse genetic assemblages also dissimilar to the canonical *Pseudomonas* operon (Figure A1, Table A2).

Conserved domain analysis of NirS homologs in this study suggests that while the C2 and NirS functional domains frequently co‐occur in nitrite reductases, the inclusion of C1 in the ORF appears extremely rare and limited to Chloroflexi. A lineage‐specific fusion of multiple gene domains could explain this novel C1‐C2‐NirS arrangement. Different evolutionary histories among the domain subunits within Chloroflexi would provide evidence for an ancestral horizontal acquisition and fusion event.

To compare the evolutionary histories of each domain in the enzyme ORF, and to determine if the different domains have different ancestry, maximum‐likelihood domain trees were reconstructed independently for C1, C2, and the NirS‐specific domain (see Methods).

**Figure 4:**
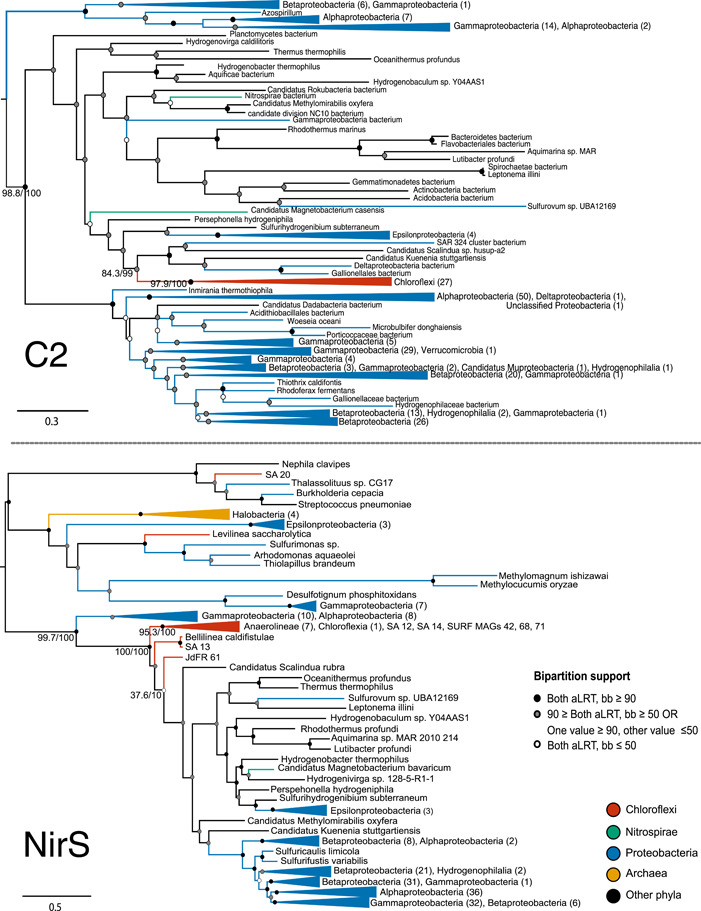
Phylogenetic trees for C2 and NirS domains. Phylogenetic analysis of C2 domain homologs (above) places the Chloroflexi within a diverse clade including Epsilonproteobacteria, Aquificae, Bacteroidetes, and Planctomycetes; this clade is sister to a broad radiation of Alpha‐, Beta‐, and Gammaproteobacteria. Analysis of NirS domain homologs (below) places the Chloroflexi within the clade dominated by Alpha‐, Beta‐, and Gammaproteobacteria, which also contains members of the Bacteroidetes and Aquificales. The overall taxonomic representation for the domains is similar. Support values for selected bipartitions are labeled (aLRT/bb). Support for other nodes is indicated with the following color scheme: Strong support with both values ≥90 (black); weak support with both values ≤50 (white); intermediate support with one or both values between 50 and 90 (gray); conflicting support, with one value ≤50 and the other ≥90 (gray)

**Figure A2:**
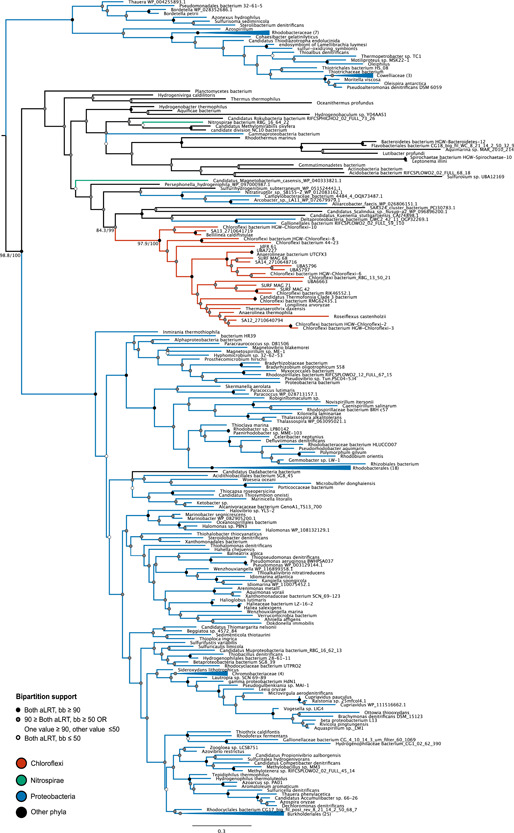
Expanded C2 domain tree. Phylogenetic analysis of C2 domain homologs places the Chloroflexi within a diverse polyphyletic clade including Epsilonproteobacteria, Aquificae, Bacteroidetes, and Planctomycetes; this clade is sister to a broad radiation of Proteobacteria. Support values for selected bipartitions are labeled (aLRT/bb). Support for other nodes is indicated with the following color scheme: Strong support with both values ≥90 (black); weak support with both values ≤50 (white); intermediate support with one or both values between 50 and 90 (gray); conflicting support, with one value ≤50 and the other ≥90 (gray)

**Figure A3:**
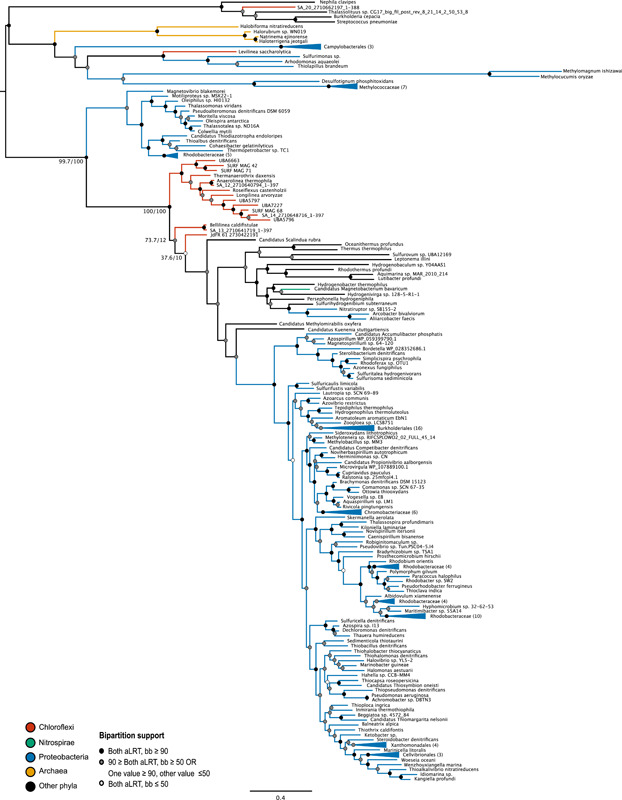
Expanded NirS domain tree. Phylogenetic analysis of NirS domain homologs places the Chloroflexi within a polyphyletic clade dominated by Alpha‐, Beta‐, and Gammaproteobacteria. Support values for selected bipartitions are labeled (aLRT/bb). Support for other nodes is indicated with the following color scheme: Strong support with both values ≥90 (black); weak support with both values ≤50 (white); intermediate support with one or both values between 50 and 90 (gray); conflicting support, with one value ≤50 and the other ≥90 (gray)

Domain phylogenies indicate similar taxonomic distributions for the C2 and NirS domains (Figure 4). The relative placement of Chloroflexi sequences varies slightly between domain trees: For the C2 domain tree, Chloroflexi sequences are monophyletic within a larger clade comprising polyphyletic sequences including members of the Aquificae, Bacteroidetes, Epsilonproteobacteria, and Spirochaetia; this clade places sister to a large group dominated by Alpha‐, Beta‐, and Gammaproteobacteria (expanded tree available as Figure A2). In the NirS domain tree, Chloroflexi place basally in a clade shared with both polyphyletic sequences and the large radiation of Proteobacterial sequences (expanded tree available as Figure A3).

**Figure A4:**
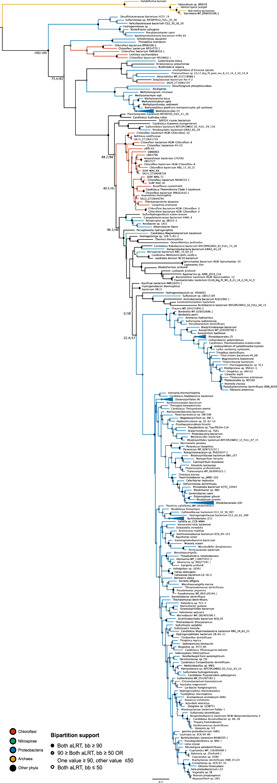
Subsampled C2 domain tree. Phylogenetic analysis of C2 domain homologs, subsampled to contain only taxa with both C2 and NirS domains in the nitrite reductase ORF, places the largest clade of Chloroflexi as sister to a polyphyletic group including a large group of Alpha‐, Beta‐, and Gammaproteobacteria. Support values for selected bipartitions are labeled (aLRT/bb). Support for other nodes is indicated with the following color scheme: Strong support with both values ≥90 (black); weak support with both values ≤50 (white); intermediate support with one or both values between 50 and 90 (gray); conflicting support, with one value ≤50 and the other ≥90 (gray). ORF, open reading frame

**Figure A5:**
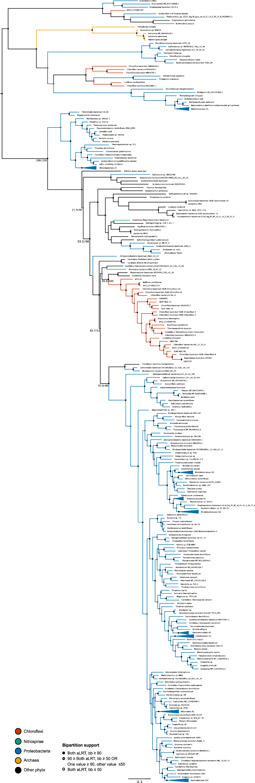
Subsampled NirS domain tree. Phylogenetic analysis of NirS domain homologs, subsampled to contain only taxa with both C2 and NirS domains in the nitrite reductase ORF, places the largest clade of Chloroflexi as sister to a large radiation of Proteobacteria; these groups are nested within a diverse polyphyletic group. Support values for selected bipartitions are labeled (aLRT/bb). Support for other nodes is indicated with the following color scheme: Strong support with both values ≥90 (black); weak support with both values ≤50 (white); intermediate support with one or both values between 50 and 90 (gray); conflicting support, with one value ≤50 and the other ≥90 (gray). ORF, open reading frame

C2 and NirS domain trees reconstructed exclusively from ORFs containing both domains produce similar topologies, albeit with slightly different placement of these major groups of taxa (Figures A4 and A5). Interestingly, the subsampling inverts the placements of the Chloroflexi with respect to the largest Proteobacterial group, as compared with the unsampled trees. This change suggests that sampling and phylogenetic noise are likely responsible for the observed differences in the C2 and NirS domain phylogenies. Additionally, there are notable differences in placement among subclades within the Proteobacteria, and low bipartition support for these subclades for the C2 tree, suggesting that patterns unrelated to Chloroflexi evolution may be polarizing the relative placements of groups in the tree. This lack of robustness caused by alternative sampling, combined with poor support values within the polyphyletic clade or between this clade and the Proteobacteria, suggests that the differences in tree topology may be artifactual, and not reflective of gene reticulation events.

**Figure 5:**
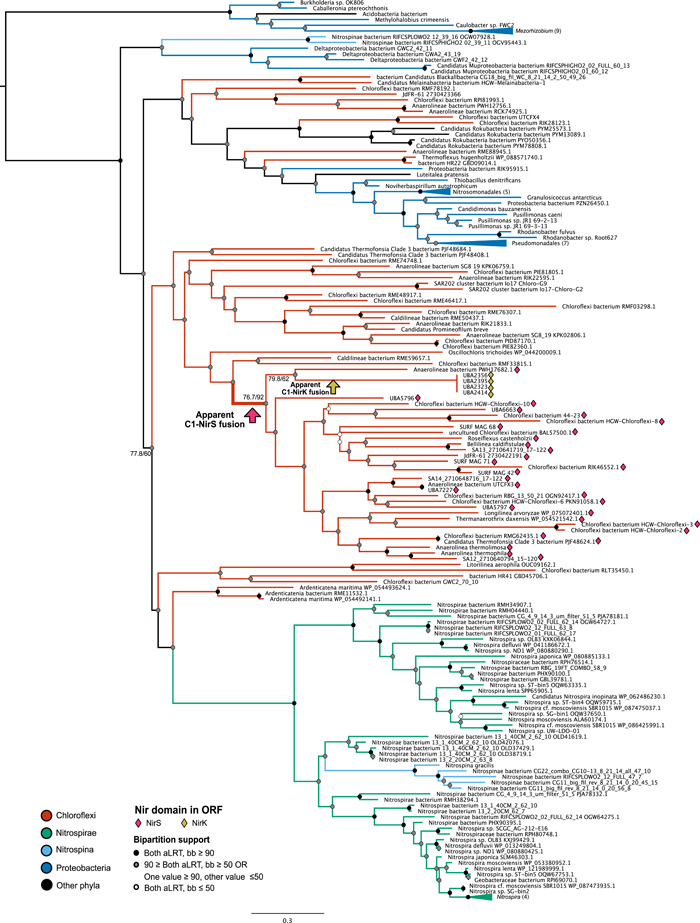
Phylogenetic tree for C1 domain. A phylogenetic tree for the C1 domain—with no genus‐level filter and inclusion of more distant hits (see Methods)—indicates a limited taxonomic distribution of the domain. The largest group of sequences in Chloroflexi places sister to domains found in Nitrospirae, Nitrospinae, and Deltaproteobacteria. Within this clade, the branch along which C1 is inferred to have fused into nitrite reductase genes in Chloroflexi is labeled. C1 homologs that co‐occur in ORFs with nitrite reductase are indicated with magenta diamonds (NirS) or yellow diamonds (NirK). Support values for selected bipartitions are labeled (aLRT/bb). Support for other nodes is indicated with the following color scheme: Strong support with both values ≥90 (black); weak support with both values ≤50 (white); intermediate support with one or both values between 50 and 90 (gray); conflicting support, with one value ≤50 and the other ≥90 (gray). ORF, open reading frame

The inferred phylogeny for C1 shows a much different evolutionary history than the other two domains (Figure 5). In contrast to the domain trees for C2 or NirS, the C1 tree shows sequences from Nitrospirae and Nitrospinae grouping together within a large clade of Chloroflexi C1 domains. Additional Chloroflexi sequences group with a small number of more distantly related Proteobacteria. However, the placement and taxonomic representation of Proteobacteria in the C1 tree is different from that seen in the other domain trees.

The majority of ORFs represented in the C1 domain tree contain the C1 domain homolog either as a free cytochrome or as one of multiple cytochrome‐type or cytochrome superfamily domains. In rare or isolated cases, C1 homologs co‐occur in ORFs with membrane or structural protein domains (Table A3). The ORF containing the C1 homolog was annotated as a NOR in several members of the Nitrospirae and one Geobacteraceae genome (Table A3); domain analysis of these genes yielded limited additional data, but representative sequences showed detectable sequence similarity to *Pseudomonas norC* genes.

**Figure A6:**
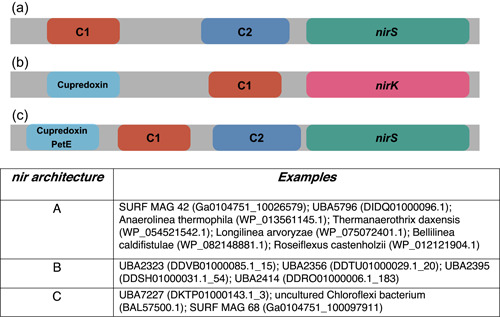
Various nitrite reductase architectures within Chloroflexi. Three different open reading frame types including a C1 cytochrome and a nitrite reductase domain appear in surveyed Chloroflexi. The most commonly seen gene features C1, C2, and cytochrome‐dependent *nirS* (a); however, C1 is also seen in ORFs with cupredoxin and copper‐dependent nitrite reductase (*nirK*) domains (b). A limited number of Chloroflexi genomes also contain ORFs placing C1, C2, and *nirS* together with an N‐terminal cupredoxin, plastocyanin (PetE), or similar copper‐containing domain (c). ORF, open reading frame

The occurrence of C1 domain homologs within predicted nitrite reductase genes is restricted to the Chloroflexi. The majority of these C1‐containing *nir* genes have the cytochrome‐type NirS domain; however, a small number of Chloroflexi MAGs contain an ORF pairing the C1 cytochrome with the copper‐type NirK domain instead. This NirK ORF also included an N‐terminal cupredoxin/plastocyanin domain. As this fusion is only apparent within a small number of MAGs, which are identical across the length of the analyzed ORF, this may represent an assembly artifact. However, several of the *nir* genes that contained a C1 homolog and a cytochrome‐type NirS (not copper‐type NirK) also contained cupredoxins or other copper‐containing domains (Figure A6).

The distinct phylogeny and taxonomic distribution of C1, as compared with C2 and NirS domains, strongly suggest that the C1‐C2‐NirS domain structure observed in Chloroflexi is the result of a fusion of the C1 domain with a horizontally‐acquired *nir* gene containing the C2 and NirS domains. Topology and extant taxon sampling of these gene trees does not allow us to reliably infer the donor lineage of this transfer. However, the C2‐NirS architecture—or similar arrangements of functional domains—is widespread among members of the Alpha‐, Beta‐, and Gammaproteobacteria; additionally, gene trees for C2 and NirS place the Chloroflexi that also contain the C1 domain within (Figure 4) or as sister to (Figures A4 and A5) Proteobacterial groups, inconsistent with species tree placements for these phyla. These data suggest the Proteobacteria as a possible donor group for the C2‐NirS domains. Additionally, it appears that Chloroflexi may have been the source for an independent transfer of the free C1 domain into Nitrospirae and Nitrospinae.

### 3.2 C1‐C2‐NirS domain architecture is unique to Chloroflexi

Though putative homologs exist independently for the constituent C1 and C2‐NirS regions, respectively, these hits reflect different cytochrome or cytochrome‐type nitrite reductases (largely in Proteobacteria, Nitrospirae, and Nitrospinae). The full C1‐C2‐NirS architecture appears unique to Chloroflexi and is not observed in other groups. Querying NCBI's nonredundant environmental database (env‐nr) with the full ORF from SURF MAG 42 did not identify additional examples of the full gene construct. While several hits were identified that reflected putative homology to the joint C2‐NirS domains, none of these included the C1 domain as well. An independent search of the env‐nr database using the C1 domain as a query returned few overall hits. While some of these putative C1 homologs were identified in ORFs containing additional cytochrome‐type enzyme superfamily domains or subunits, none co‐occurred with NirS or NirK domains. These data suggest that there is little to no missing diversity of the Chloroflexi‐type chimeric nitrite reductase in existing metagenomes.

Attempts to visualize the full enzyme structure using homology modeling (Bienert et al., 2017; A. Waterhouse et al., 2018) were unsuccessful; structural models were only able to predict a close match for the conserved C2‐NirS region of the putative gene. Efforts to independently model the C1 structure could not recover predicted QMEAN scores above −4.50 (Benkert et al., 2011). The poor scores may reflect the relatively short length of the cytochrome coding region. However, the Chloroflexi *nirS* gene sequence does retain several conserved residues present in the crystal structure of *P. aeruginosa* NirS. In *P. aeruginosa* NirS, His51, and Met88 coordinate heme *c*; His182 coordinates heme d1; and His327 and His369 are believed to stabilize the active site nitrite anion (Maia & Moura, 2014; Rinaldo et al., 2011). Corresponding residues are conserved within the C2 (His65, Met125) and NirS alignments (His46, His239, His300) for the Chloroflexi NirS ORF; interestingly, the residue corresponding to His327 (His239) is not universally conserved, though it is conserved among Chloroflexi with the novel NirS architecture.

### 3.3 Expansion of eNOR diversity

**Figure A7:**
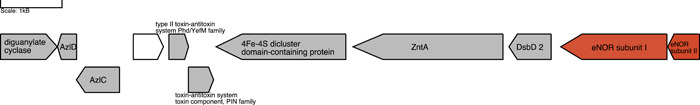
eNOR subunit gene neighborhood. A gene neighborhood showing the 20,000 base pair region adjacent to eNOR subunits in SURF MAG 42. Sequence analysis of subunit I (NCBI protein accession RJP50323.1) reflects a conserved glutamine substitution characteristic of the eNOR subfamily. Both proposed subunits of eNOR—the heme‐copper cytochrome‐containing subunit I, and the cupredoxin‐containing subunit II—appear in the MAG. No other denitrification genes appear in the neighborhood. MAG, metagenome‐assembled genome; NCBI, National Center for Biotechnology Information; SURF, Sanford Underground Research Facility

**Figure A8:**
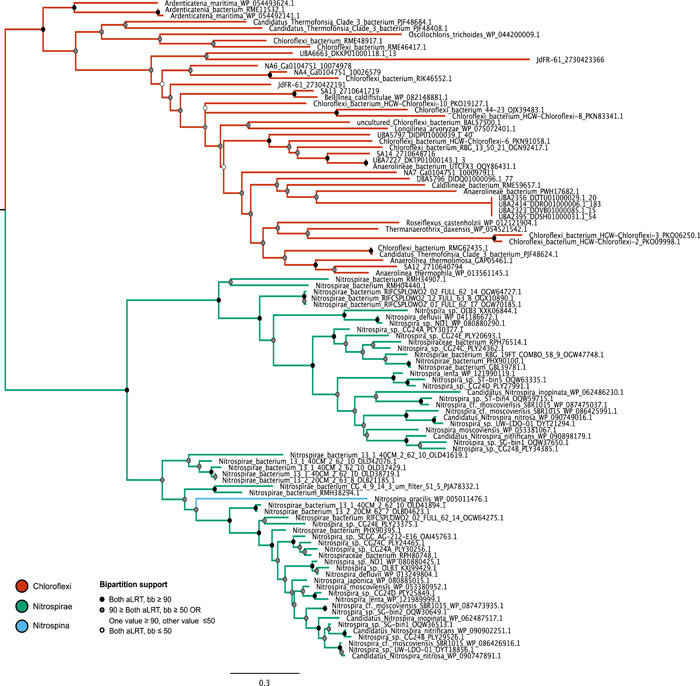
Preliminary C1 domain tree. A preliminary phylogenetic tree for C1, including only sequences with *E* ≤ 10^−10^, contains very few overall taxa. The tree contains only members of the Chloroflexi, members of the Nitrospirae, and *Nitrospina gracilis*; the sampling depth does not recover an outgroup for these sister groups. Support for bipartitions is indicated with the following color scheme: Strong support with both values ≥90 (black); weak support with both values ≤50 (white); intermediate support with one or both values between 50 and 90 (gray); conflicting support, with one value ≤50 and the other ≥90 (gray)

Notably, the majority of genomes with the unique C1‐C2‐NirS structure do not appear to contain a NOR gene (*nor*). Though the absence of the *nor* gene in genomes with *nirS* is not unprecedented, previous genomic surveys suggest it is relatively uncommon, and the toxicity of the product of Nir (NO) makes this absence counterintuitive (Graf et al., 2014; Hendriks et al., 2000). However, analysis of the SURF MAG 42 metagenome—the originally assembled genome in which the novel *nirS* ORF was observed—did reveal the presence of an unusual *nor* homolog. Previous studies have identified the established cNOR and qNOR family enzymes (which contain cytochrome c or quinols as electron donors, respectively) in Chloroflexi, as well as a broad distribution of Proteobacteria (Hemp & Gennis, 2008; Hendriks et al., 2000; Zumft, 2005). However, the predicted NOR in SURF MAG 42 included an active site glutamine substitution characteristic of eNOR (Hemp & Gennis, 2008; Hemp et al., 2015; Table A4). SURF MAG 42 contains both proposed subunits (Hemp & Gennis, 2008) of eNOR; the diagnostic subfamily substitution is within the heme‐copper cytochrome‐containing subunit I, and the cupredoxin‐containing subunit II is immediately upstream in the MAG (Figure A7). eNOR has been previously described in Archaea (Hemp & Gennis, 2008) and at least one isolated Anaerolineales bacterium (Hemp et al., 2015).

**Figure 6:**
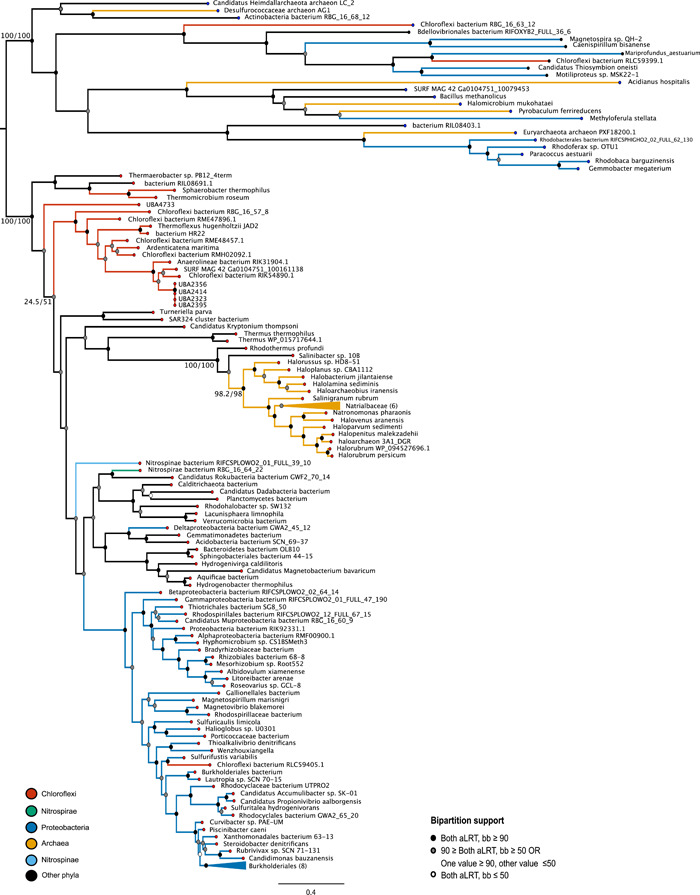
*eNOR* gene tree. A phylogenetic tree of homologs to the nitric oxide reductase from SURF MAG 42 reveals an expanded diversity of putative *eNOR* subunit I homologs in not only Archaea and Chloroflexi, but also Proteobacteria and other diverse phyla. Putative *eNOR* sequences (red tips) have the characteristic Gln‐323 in the alignment; outgroup sequences (blue tips) have Tyr‐323 (oxygen reductase superfamily) or other substitutions. Support values for selected bipartitions are labeled (aLRT/bb). Support for other nodes is indicated with the following color scheme: Strong support with both values ≥90 (black); weak support with both values ≤50 (white); intermediate support with one or both values between 50 and 90 (gray); conflicting support, with one value ≤50 and the other ≥90 (gray). MAG, metagenome‐assembled genome; SURF, Sanford Underground Research Facility

Phylogenetic analysis indicates the presence of *eNOR* in an expanded diversity of genomes (Figure 6). Previous studies have described eNOR in *Natronomonas*; these data indicate a cluster of *eNOR* genes throughout other Halobacteria as well. Additional putative *eNOR* genes appear in multiple members of Anaerolineales, as well as other Chloroflexi, and in many members of the Alpha‐, Beta‐, Gamma‐, and Deltaproteobacteria. Many of these putative *eNOR* subunit homologs appear to have been misannotated or mislabeled as cytochrome *c* oxidase genes, likely because of the structural similarity of the heme–copper cytochrome region (Hemp & Gennis, 2008; Marchler‐Bauer et al., 2015).

## 4 DISCUSSION

The phylogenetic analyses of *nirS* and *eNOR* ORFs in Chloroflexi suggest that subsurface ecosystems may harbor an under‐described diversity of denitrification enzymes, which may reflect adaptations to the unique challenges of nutrient cycling within these environments. More broadly, a deeper understanding of the ecological extent of microbial denitrification has important implications for basic and applied microbial ecology. The reduction of fixed nitrogen species plays a crucial role in global nitrogen cycling and is also an essential component of smaller‐scale systems, such as those associated with agricultural or waste treatment (Butterbach‐Bahl & Dannenmann, 2011; H. Lu et al., 2014). The discovery and characterization of novel variants of genes such as *nirS* and *eNOR* may therefore pave the way for future biotechnological applications.

Although the C2 and NirS domains do not have identical evolutionary histories or distributions, the taxonomic representation of these groups is very similar, and the presence of the paired C2‐NirS domains in cytochrome‐type nitrite reductases appears broadly throughout the Proteobacteria. In contrast, the taxonomic distribution and phylogeny of the C1 domain tree are strikingly different than that of the other domains in the nitrite reductase ORF. Combined with the apparent absence of a full C1‐C2‐NirS ORF in any taxonomic group other than Chloroflexi, these data suggest that the C1 cytochrome was likely incorporated into *nirS* in a gene fusion event within Chloroflexi, following HGT. As there is no evidence of the C2‐NirS ORF in Chloroflexi without the fused C1 domain present, the fusion probably occurred very soon after the acquisition of the C2‐NirS region and may be necessary for the function of the gene in Chloroflexi.

In *P. aeruginosa*, cytochrome c551—encoded by *nirM*—is the electron donor for the adjacent cytochrome *cd1 nirS* (Philippot, 2002; Zumft, 1997). Homology searches do not identify any regions within SURF MAG 42 with significant similarity to *Pseudomonas nirM*, but C1 is identified as a putative member of the cytochrome c551/552 family. It is, therefore, possible that C1, though divergent from Proteobacterial *nirM*, serves a similar redox role for the cytochrome *cd1* now within the same ORF.

Interestingly, putative homologs of C1 cytochrome domains were found in some Chloroflexi genomes in ORFs containing *nirK*, not *nirS* (Figures 5 and A6). Though NirS and NirK are functionally equivalent, the two enzymes do not show a shared evolutionary origin and are often—though not always—mutually exclusive among known denitrifier genomes (Graf et al., 2014; Jones et al., 2008). Unlike the cytochrome‐containing NirS, NirK is a copper‐type enzyme. The co‐occurrence of cytochrome *c* domains in ORFs with the copper‐type *nirK* has been identified in rare instances in Proteobacteria, and noted as surprising, given the cupredoxin‐like fold of the NirK enzyme (Bertini et al., 2006). Similarly surprising is the inverse relationship revealed in the C1 domain tree: Several Chloroflexi ORFs contain a cupredoxin or similar copper‐containing domain N‐terminal to the C1‐C2‐NirS architecture (Figure A6, Table A3). The co‐occurrence of C1 with both cytochrome‐ and copper‐dependent Nir domains suggests a general evolutionary trend within Chloroflexi to incorporate this cytochrome into denitrification ORFs. This distribution pattern raises the possibility that the C1‐type cytochrome may serve an important but generalized role in nitrite reduction—regardless of the evolutionary history or genetic profile of the nitrite reduction domain itself.

The apparent absence of a *nor* homolog in the majority of genomes with the C1‐*nirS* fusion is unexpected. Beyond providing downstream redox capacity, NOR provides an efficient means of reducing and detoxifying NO, the highly cytotoxic product of NirS. It is not unprecedented for bacterial genomes to harbor a *nir* gene without a *nor* gene, particularly for organisms with *nirK* (Graf et al., 2014; Heylen et al., 2007). This *nir–nor* mismatch is much rarer for putative denitrifiers with *nirS*, representing fewer than 4% of genomes in a recent survey—but a small number of surveyed bacteria do, interestingly, appear to harbor *nirS* without also harboring *cNOR* or *qNOR* (Graf et al., 2014; Heylen et al., 2007). To our knowledge, however, *eNOR* has not been included in such analyses of the genomic correlation between nitrite reductases and NORs. The phylogenetic evidence for diverse *eNOR* homologs suggests likely undocumented or underexplored diversity for divergent NORs. The diversity and function of cNOR and qNOR are fairly well‐established. However, divergent enzymes such as eNOR and sNOR are less extensively documented and may not be accurately distinguished from broader oxygen reductase superfamily members in genomic or metagenomic analyses.

Cytochrome *c* proteins function as electron transfer proteins in anaerobic respiration and are often fused to redox enzymes to allow electron passage (Bertini et al., 2006). It is not surprising, therefore, to find cytochrome *c*‐containing subunits in frame with nitrite reductase. NirS itself is cytochrome‐dependent (Bertini et al., 2006). However, the unusual addition of the upstream cytochrome domain (C1) may reflect additional redox requirements or capacity. It is also possible that the inclusion of this construct could be linked to the conspicuous absence of NOR enzymes in several MAGs containing a NirS ORF with the C1 fusion. NO reduction can be cytochrome‐dependent; the well‐studied cNORs contain a membrane‐anchored cytochrome *c* (Hemp & Gennis, 2008). Further, the C1 domain tree recovers ORFs in the Nitrospirae that contain C1 homologs and are annotated as NORs, with detectable similarity to Proteobacteria NOR subunits. It is therefore possible that the inclusion of a C1 domain in *nir* genes within genomes lacking *eNOR* reflects some generalized NOR‐like role in detoxification of the cytotoxic product of NirS. Additionally, while the presence of NirS suggests an active denitrification pathway, and the NirS domain tree reflects the homology between this domain and NirS from known denitrifying groups, the possibility remains that this group of Chloroflexi do not perform denitrification, and instead use this gene product for a different metabolic function, potentially enabled or constrained by the C1 domain. The genetic diversity observed within C1‐C2‐NirS gene neighborhoods—varying both from the genetic makeup of a canonical denitrification operon, and between different Chloroflexi MAGs—may reflect such functional flexibility. Shared or convergent functions could also be recovered among these diverse neighborhoods. The analyzed gene neighborhoods suggest some conserved functionality between different genomes from varying environmental sources and samples; for example, the presence of heme–copper oxidase subunits, molybdenum cofactor enzymes, and NADB Rossman superfamily proteins (Tables A1 and A2). However, without expanded homology analyses and experimental validation, it is impossible to infer whether apparent similarities or differences reflect biologically‐meaningful patterns of inheritance or function, or are simply artifactual.

A divergent or generalized role is also possible for the eNOR homologs. Models indicate that eNOR (unlike related NORs cNOR, qNOR, sNOR, and gNOR) has a proton channel, and therefore the capacity for proton pumping (Hemp & Gennis, 2008); therefore, this gene product may serve a key electrogenic role, whether reducing NO or alternative substrates. Experimental validation would be necessary to determine if the novel Chloroflexi‐associated NirS performs differently than canonical NirS in vivo, and if NirS and eNOR perform targeted denitrification or nonspecific detoxification or proton pumping. This study, therefore, suggests a promising direction for future investigations.

The divergent denitrification enzymes described above may or may not reflect different metabolic strategies in situ. But the identification of both a novel *nirS* ORF and an expanded diversity of eNOR enzymes suggests that the existing understanding of denitrification may underestimate the genetic diversity and ecological distribution of constituent enzymes. This may be especially true in deep subsurface biomes, such as those from which several Chloroflexi analyzed in this study were isolated. These systems have garnered increasing attention in recent years; extensive evidence supports the existence of dynamic, diverse microbial subsurface ecosystems with the metabolic potential to influence global biogeochemical cycles (Hug et al., 2013; Magnabosco et al., 2018; Momper & Jungbluth, 2017; Osburn et al., 2014, 2019). Chloroflexi are frequently cited as well‐represented members of deep sediment and aquifer systems, where they play key roles in carbon cycling dynamics (Hug et al., 2013; Kadnikov et al., 2020; Momper & Jungbluth, 2017; Momper & Kiel Reese, 2017). But Chloroflexi are known to also harbor diverse nitrogen metabolisms (Denef et al., 2016; Hemp et al., 2015; Spieck et al., 2020), and previous studies have linked subsurface Chloroflexi to denitrification pathway genes such as nitrous oxide reductase (*nos*) (Hug et al., 2016; Momper & Jungbluth, 2017; Sanford et al., 2012). The role of Chloroflexi in subsurface nitrogen cycling—as well as the scope of subsurface microbial nitrogen dynamics at large—requires further investigation.

## CONFLICT OF INTEREST

None declared.

## ETHICS STATEMENT

None required.

## AUTHOR CONTRIBUTIONS

**Sarah L. Schwartz**: Conceptualization (supporting), Data curation (lead), Formal analysis (lead), Funding acquisition (equal), Investigation (lead), Methodology (lead), Project administration (lead), Validation (equal), Visualization (lead), Writing – original draft (lead), Writing – review & editing‐Lead. **Lily M. Momper**: Conceptualization (lead); Data curation (supporting); Investigation (supporting); Methodology (supporting); Writing – original draft (supporting). **L. Thiberio Rangel**: Data curation (supporting), Investigation (supporting), Methodology (equal), Writing – review & editing (supporting). **Cara Magnabosco**: Data curation (supporting), Investigation (supporting), Methodology (supporting). **Jan P. Amend**: Conceptualization (supporting), Data curation (supporting), Project administration (supporting), Resources (supporting), Writing – review & editing (supporting). **Gregory P. Fournier**: Conceptualization (supporting), Formal analysis (supporting), Funding acquisition (equal), Investigation (supporting), Methodology (supporting), Project administration (lead), Resources (lead), Supervision (lead), Writing – review & editing (lead).

## Data Availability

The data that support the findings of this study are openly available in figshare at https://doi.org/10.6084/m9.figshare.14515554.v2. Scripts used to automate analyses are archived in Zenodo at https://doi.org/10.5281/zenodo.5745924.

## APPENDIX 2

**Table A1:** Open reading frame descriptions for SURF MAG 42, *Pseudomonas aeruginosa* PAO1, and *Anaerolinea thermolimosa* NirS gene neighborhoods

Displayed ORF name	Expanded description	Protein ID	Source MAG/organism
Xylose isomerase‐like	Sugar phosphate isomerase/epimerase, xylose isomerase‐like TIM barrel	RJP53741.1	SURF MAG 42
LuxS	S‐ribosylhomocysteine lyase	RJP53742.1	SURF MAG 42
EEVS	2‐Epi‐5‐epi‐valiolone synthase/iron‐containing alcohol dehydrogenase	RJP53743.1	SURF MAG 42
UbiG	Ubiquinone biosynthesis *O*‐methyltransferase/bifunctional 2‐polyprenol‐6‐hydroxyphenol	RJP53744.1	SURF MAG 42
UbiA	ubiA family prenyltransferase	RJP53745.1	SURF MAG 42
MhpC	Alpha/beta hydrolase/pimeloyl‐ACP methyl ester carboxylesterase	RJP53746.1	SURF MAG 42
UDG‐like	Uracil‐DNA glycosylase	RJP53748.1	SURF MAG 42
BaeS	HAMP domain‐containing protein/signal transduction kinase	RJP53750.1	SURF MAG 42
*N*‐acetyltransferase	GNAT family *N*‐acetyltransferase	RJP53751.1	SURF MAG 42
PrSA	Ribose‐phosphate pyrophosphokinase/phosphoribosylpyrophosphate synthetase	RJP53766.1	SURF MAG 42
PolX	DNA polymerase/3ʹ−5ʹ exonuclease	RJP53755.1	SURF MAG 42
YdcZ	DMT family transporter	RJP53756.1	SURF MAG 42
Putative PMT 2 superfamily	Dolichyl‐phosphate‐mannose‐protein mannosyltransferase	RJP53757.1	SURF MAG 42
Acetyl‐CoA dehydrogenase	Probable acyl‐CoA dehydrogenase	AAG03897.1	PAO1
nirN	Probable c‐type cytochrome	AAG03898.1	PAO1
SUMT	Probably uroporphyrin‐III c‐methyltransferase	AAG03899.1	PAO1
nirJ	Heme d1 biosynthesis protein NirJ	AAG03900.1	PAO1
nirH	Heme d1 biosynthesis protein NirH	AAG03901.1	PAO1
nirG	Heme d1 biosynthesis protein NirG/probable transcriptional regulator	AAG03902.1	PAO1
nirL	Heme d1 biosynthesis protein NirL	AAG03903.1	PAO1
nirD	Heme d1 biosynthesis protein nirD/probable transcriptional regulator	AAG03904.1	PAO1
nirF	Heme d1 biosynthesis protein NirF	AAG03905.1	PAO1
nirC	Probable c‐type cytochrome precursor	AAG03906.1	PAO1
nirM	Cytochrome c‐551 precursor	AAG03907.1	PAO1
nirQ	Regulatory protein NirQ	AAG03909.1	PAO1
Heme‐Cu oxidase III	Heme–copper oxidase, subunit III	AAG03911.1	PAO1
norC	Nitric‐oxide reductase subunit C	AAG03912.1	PAO1
norB	Nitric‐oxide reductase subunit B	AAG03913.1	PAO1
norD	Probable denitrification protein NorD	AAG03914.1	PAO1
Dnr	Transcriptional regulator DNR	AAG03916.1	PAO1
rsmY	Regulatory RNA RsmY/probable transcriptional regulator	AAG03917.1	PAO1
CynR	Provisional; DNA‐binding transcriptional regulator	AAG03917.1	PAO1
YiiM	Uncharacterized conserved protein/MOSC (molybdenum cofactor sulfurase C‐terminal)domain‐containing protein	AAG03918.1	PAO1
ArgD	Acetylornithine/succinyldiaminopimelate/putrescin e aminotransferase	AAG03919.1	PAO1
TrpB‐like	TrpB‐like pyridoxal‐phosphate dependent enzyme	GAP05449.1	*Anaerolinea thermolimosa*
Gph	Phosphoglycolate phosphatase; haloacid dehalogenase family hydrolase	GAP05450.1	*Anaerolinea thermolimosa*
DR1245‐like	Uncharacterized, YbjN domain‐containing protein; possible type III secretion system chaperone protein	GAP05451.1	*Anaerolinea thermolimosa*
peptidase C39‐like	Peptidase C39‐like and TPR domain‐containing protein	GAP05452.1	*Anaerolinea thermolimosa*
DUF2568	Hypothetical protein; YrdB family; DUF2568	GAP05453.1	*Anaerolinea thermolimosa*
DUF4388	Hypothetical protein; DUF4388	GAP05454.1	*Anaerolinea thermolimosa*
Srp102	ATP/GTP‐binding protein; GTPase, signal recognition particle receptor subunit beta	GAP05455.1	*Anaerolinea thermolimosa*
MhpC	Predicted alpha/beta hydrolase; pimeloyl‐ACP methyl ester carboxylesterase	GAP05456.1	*Anaerolinea thermolimosa*
glmU	*N*‐acetylglucosamine‐1‐phosphate uridyltransferase; left‐handed parallel beta‐helix domain	GAP05458.1	*Anaerolinea thermolimosa*
Cytochrome cd1	Protein containing cytochrome D1 heme domain; nitrite reductase	GAP05460.1	*Anaerolinea thermolimosa*
Cytidylate kinase‐like	Cytidylate kinase‐like family protein; NK superfamily	GAP05462.1	*Anaerolinea thermolimosa*
Glycosyl hydrolase catalytic core	Glycosyl hydrolases superfamily	GAP05463.1	*Anaerolinea thermolimosa*
Protoheme IX farnesyltransferase	Provisional protoheme IX farnesyltransferase; cytochrome oxidase assembly protein superfamily	GAP05464.1	*Anaerolinea thermolimosa*
SURF1	SURF1 superfamily; similar to yeast cytochrome oxidase assembly protein SHY1	GAP05465.1	*Anaerolinea thermolimosa*
SCO	SCO1/SenC/PrrC; synthesis of cytochrome c oxidase family	GAP05467.1	*Anaerolinea thermolimosa*
Heme Cu oxidase III	Heme–copper oxidase subunit III	GAP05468.1	*Anaerolinea thermolimosa*
COX4 pro	Cytochrome *c* oxidase subunit IV family protein; prokaryotic cytochrome C oxidase subunit IV	GAP05469.1	*Anaerolinea thermolimosa*
Heme Cu oxidase I	Heme–copper oxidase subunit I	GAP05470.1	*Anaerolinea thermolimosa*
CyoA	Cytochrome *c* oxidase, subunit II; cytochrome *c*	GAP05471.1	*Anaerolinea thermolimosa*

Abbreviations: MAG, metagenome‐assembled genome; SURF, Sanford Underground Research Facility.

**Table A2:** Open reading frame (ORF) descriptions for Chloroflexi bacterium 44‐23 and SURF MAG 71 NirS gene neighborhoods

Displayed ORF name	Expanded description	Protein ID	Source organism
Y1 Tnp	Hypothetical protein; Y1 Tnp superfamily, transposase IS200‐like	OJX39478.1	Chloroflexi bacterium 44‐23
DEAD/DEAH	DEAD/DEAH box helicase; type I restriction endonuclease subunit R	OJX39479.1	Chloroflexi bacterium 44‐23
PDDEXK‐like	Hypothetical protein; PDDEXK nuclease‐like superfamily	OJX39480.1	Chloroflexi bacterium 44‐23
MobA	Hypothetical molybdenum cofactor guanylyltransferase	OJX39484.1	Chloroflexi bacterium 44‐23
DUF2298	Hypothetical protein; uncharacterized membrane protein DUF2298 superfamily; helix‐hairpin‐helix motif	OJX39485.1	Chloroflexi bacterium 44‐23
PMT2/PA14	Hypothetical protein; PA14 domain; Dolichyl‐phosphate‐mannose‐protein mannosyltransferase superfamily	OJX39484.1	Chloroflexi bacterium 44‐23
NADB Rossmann	Hypothetical protein; NADB Rossmann superfamily; Rossmann‐fold NAD(P)(+)‐binding proteins	OJX39487.1	Chloroflexi bacterium 44‐23
PMT2	Hypothetical protein; Dolichyl‐phosphate‐mannose‐protein mannosyltransferase	OJX39488.1	Chloroflexi bacterium 44‐23
MFS MOT1	Hypothetical protein; putative sulfate/molybdate transporter, MFS superfamily	RJP52521.1	SURF MAG 71
CopZ	Copper chaperone CopZ; heavy‐metal‐associated domain‐containing protein	RJP52522.1	SURF MAG 71
DsbD 2	Hypothetical protein; DsbD 2 cytochrome c biogenesis protein transmembrane region; cupredoxin superfamily‐containing protein	RJP52523.1	SURF MAG 71
P‐type ATPase	Heavy metal translocating P‐type ATPase, Cu‐like	RJP52525.1	SURF MAG 71
HMA	Heavy metal transporter, HMA superfamily	RJP52526.1	SURF MAG 71
CsoR‐like	Metal‐sensitive transcriptional regulator; TthCsoR‐like DUF156	RJP52527.1	SURF MAG 71
S2P‐M50‐like	Site‐2 protease family protein; zinc metalloproteases	RJP52530.1	SURF MAG 71
GalE	UDP‐glucose 4‐epimerase; NADB Rossmann superfamily	RJP52531.1	SURF MAG 71
Fes	Hypothetical protein; Fes superfamily; Enterochelin esterase or related enzyme	RJP52532.1	SURF MAG 71

Abbreviations: MAG, metagenome‐assembled genome; SURF, Sanford Underground Research Facility.

**Table A3:** Domain families in ORFs with C1 domain homolog but no NirS domain homolog

Domain name	Brief description	Accession(s)
CyoA	Cytochrome/quinol oxidase	RME74748.1
COX IV	Prokaryotic cytochrome c subunit IV	PKB63614.1; PIQ26724.1
tynA	Primary‐amine oxidase	WP_095041976.1
CxxCH_TIGR02603	Putative heme‐binding domain	WP_095041976.1; OUC09162.1; OGW07928.1; OGP31126.1; OGV95443.1; OGP47454.1; OGP11994.1; OG149337.1; OYT21294.1; OLD38719.1; OLB21185.1
Cupredoxin	N/A	UBA5757_DIDP01000039.1_40
Caa3_CtaG	Cytochrome c oxidase caa3 assembly factor	PYQ29527.1
PRK10856	Cytoskeleton protein RodZ	OQY877.1
EnvC	Septal ring factor, activator of murein hydrolases	RMF78192.1
PRK03735	Provisional cytochrome b6	RME88945.1
DMSOR_beta‐like	DMSO reductase beta subunit	RME88945.1
FlpD	Methyl‐viologen‐reducing	RME88945.1
	hydrogenase, beta subunit	
TTQ_mauG	tryptophan tryptophylquinone biosynthesis enzyme MauG	RIK95915.1
Heme_Cu_Oxidase_I	Heme‐copper oxidase subunit I	WP_073101466.1
PRK14486	Putative bifunctional cbb3‐type cytochrome c oxidase subunit II	OYW96265.1
YccC	Uncharacterized membrane protein	OLB04623.1; OAI45763.1; WP_080880425.1; KXJ99429.1
FTR1	Iron permease	WP_053380952.1
EcfT	T component of ECF‐type transporters	WP_090902251.1
ArsB_NhaD_permease	Anion permease	OYT18856.1; WP_090747891.1

Abbreviation: ORF, open reading frame.

**Table A4:** Heme–copper superfamily active site (modified from Hemp & Gennis, 2008)

Enzyme	Active‐site residue	Predicted no. of proton channels	Present in
Oxygen reductase	Y	0–2	All domains; varies by enzyme subfamily
cNOR	E	0	Proteobacteria (Shiro, 2012; Hendriks, 2000)
qNOR	E	0	Proteobacteria, Cyanobacteria, Firmicutes, Archaea (Shiro, 2012; Hemp & Gennis, 2008; Heylen et al., 2007; Hendriks et al, 2000)
sNOR	N	0	Betaproteobacteria, *Silicibacter, Deinococcus*, *Geobacillus* (Stein et al., 2007)
eNOR	Q	1	Archaea (Hemp & Gennis, 2008, this study), Chloroflexi (Ward, McGlynn et al., 2018 this study), Proteobacteria (this study)
gNOR	D	0	*Sulfurimonas*, *Sulfurovum, Persephonella* (Sievert et al., 2008)
CuANor	N	1	*Bacillus* (Al‐Attar & De Vries, 2015; Suharti et al., 2001)

Abbreviations: cNOR, cytochrome‐type nitric oxide reductase; qNOR, quinol‐dependent nitric oxide reductases.

**Table A5:** MAG source data

Genome	BioProject	BioSample	MAG name	MAG/hit db filename	Publication
1	PRJNA355136	SAMN08499021	SURF MAG 27	NA1	Momper, Jungbluth et al. (2017)
2	PRJNA355136	SAMN08499024	SURF MAG 30	NA2	Momper, Jungbluth et al. (2017
3	PRJNA355136	SAMN08499034	SURF MAG 40	NA3	Momper, Jungbluth et al. (2017)
4^a^	PRJNA355136	SAMN08499036	SURF MAG 42	NA4	Momper, Jungbluth et al. (2017)
5	PRJNA355136	SAMN08499037	SURF MAG 43	NA5	Momper, Jungbluth et al. (2017)
6	PRJNA355136	SAMN08499065	SURF MAG 71	NA6	Momper, Jungbluth et al. (2017)
7	PRJNA355136	SAMN08499062	SURF MAG 68	NA7	Momper, Jungbluth et al. (2017)
8	PRJNA269163	SAMN06226378	JdFR‐61	JdFR61	Jungbluth et al. (2017)
9	PRJNA681409/PRJNA680468	pending release	–	SA8	–
10	PRJNA681409/PRJNA680468	pending release	–	SA9	–
11	PRJNA681409/PRJNA680468	pending release	–	SA10	–
12	PRJNA681409/PRJNA680468	pending release	–	SA11	–
13	PRJNA681409/PRJNA680468	pending release	–	SA12	–
14	PRJNA681409/PRJNA680468	pending release	–	SA13	–
15	PRJNA681409/PRJNA680468	pending release	–	SA14	–
16	PRJNA681409/PRJNA680468	pending release	–	SA15	–
17	PRJNA681409/PRJNA680468	pending release	–	SA16	–
18	PRJNA681409/PRJNA680468	pending release	–	SA17	–
19	PRJNA681409/PRJNA680468	pending release	–	SA18	–
20	PRJNA681409/PRJNA680468	pending release	–	SA19	–
21	PRJNA681409/PRJNA680468	pending release	–	SA20	–

Abbreviations: ORF, open reading frame; MAG, metagenome‐assembled genome.

^a^ SURF MAG 42 is the source genome for nirS ORF.

**Table A6:** MAG identifying data for sequences selected from Parks et al. (2017)

MAG no.		Organism name				Isolate ID		WGS ID	
1		Anaerolineaceae		bacterium		UBA1024		DCER00000000	
2		Anaerolineaceae		bacterium		UBA2178		DCVW00000000	
3		Anaerolineaceae		bacterium		UBA2274		DDWY00000000	
4		Anaerolineaceae		bacterium		UBA6073		DIXT00000000	
5		Anaerolineaceae		bacterium		UBA7644		DLIK00000000	
6		Anaerolineales		bacterium		UBA1429		DCTX00000000	
7		Anaerolineales		bacterium		UBA2181		DCVT00000000	
8		Anaerolineales		bacterium		UBA2200		DCVA00000000	
9		Anaerolineales		bacterium		UBA2317		DDVH00000000	
10		Anaerolineales		bacterium		UBA2323		DDVB00000000	
11		Anaerolineales		bacterium		UBA2356		DDTU00000000	
12		Anaerolineales		bacterium		UBA2395		DDSH00000000	
13		Anaerolineales		bacterium		UBA2414		DDRO00000000	
14		Anaerolineales		bacterium		UBA2796		DEHQ00000000	
15		Anaerolineales		bacterium		UBA6092		DIXA00000000	
16		Anaerolineales		bacterium		UBA6663		DKKP00000000	
17		Anaerolineales		bacterium		UBA6665		DKKN00000000	
18		Anaerolineales		bacterium		UBA7227		DKTP00000000	
19		Chloroflexaceae		bacterium		UBA1466		DCSM00000000	
20		Chloroflexi		bacterium		UBA2235		DDYL00000000	
21		Chloroflexi		bacterium		UBA5177		DHWR00000000	
22		Chloroflexi		bacterium		UBA5183		DHWL00000000	
23		Chloroflexi		bacterium		UBA6042		DIYY00000000	
24		Chloroflexi		bacterium		UBA6077		DIXP00000000	
25		Chloroflexi		bacterium		UBA6265		DJVD00000000	
26		Chloroflexi		bacterium		UBA6622		DJHK00000000	
27		Dehalococcoidia		bacterium		UBA1127		DCAS00000000	
28		Dehalococcoidia		bacterium		UBA1151		DBZV00000000	
29		Dehalococcoidia		bacterium		UBA1222		DBXC00000000	
30		Dehalococcoidia		bacterium		UBA2141		DCXH00000000	
31		Dehalococcoidia		bacterium		UBA2158		DCWQ00000000	
32		Dehalococcoidia		bacterium		UBA2160		DCWO00000000	
33		Dehalococcoidia		bacterium		UBA2243		DDYD00000000	
34		Dehalococcoidia		bacterium		UBA2247		DDXZ00000000	
35		Dehalococcoidia		bacterium		UBA2588		DDKW00000000	
36		Dehalococcoidia		bacterium		UBA2768		DEIS00000000	
37		Dehalococcoidia		bacterium		UBA2962		DEBG00000000	
38		Dehalococcoidia		bacterium		UBA2979		DEAP00000000	
39		Dehalococcoidia		bacterium		UBA6234		DJWI00000000	
40		Dehalococcoidia		bacterium		UBA6803		DKFF00000000	
41		Dehalococcoidia		bacterium		UBA6926		DKAM00000000	
42		Dehalococcoidia		bacterium		UBA6951		DJZN00000000	
43		Dehalococcoidia		bacterium		UBA6956		DJZI00000000	
44		Dehalococcoidia		bacterium		UBA7833		DLTP00000000	
45		Dehalococcoidia		bacterium		UBA7894		DLRG00000000	
46		Dehalococcoidia		bacterium		UBA826		DBHO00000000	
47		bacterium		UBP15		UBA6099		DIWT00000000	
48		Anaerolineaceae		bacterium		UBA4775		DHHJ00000000	
49		Anaerolineaceae		bacterium		UBA4784		DHHA00000000	
50		Anaerolineaceae		bacterium		UBA4841		DHEV00000000	
51		Anaerolineaceae		bacterium		UBA4890		DHCY00000000	
52		Anaerolineaceae		bacterium		UBA4929		DHBL00000000	
53		Anaerolineaceae		bacterium		UBA5199		DHVV00000000	
54		Anaerolineaceae		bacterium		UBA5224		DHUW00000000	
55		Anaerolineaceae		bacterium		UBA5229		DHUR00000000	
56		Anaerolineaceae		bacterium		UBA5241		DHUF00000000	
57		Anaerolineaceae		bacterium		UBA5243		DHUD00000000	
58		Anaerolineaceae		bacterium		UBA5311		DHRN00000000	
59		Anaerolineaceae		bacterium		UBA5344		DHQG00000000	
60		Anaerolineaceae		bacterium		UBA5355		DHPV00000000	
61		Anaerolineaceae		bacterium		UBA5404		DHNY00000000	
62		Anaerolineaceae		bacterium		UBA5823		DICP00000000	
63		Anaerolineales		bacterium		UBA4142		DFWE00000000	
64		Anaerolineales		bacterium		UBA4826		DHFK00000000	
65		Anaerolineales		bacterium		UBA5215		DHVF00000000	
66		Anaerolineales		bacterium		UBA5796		DIDQ00000000	
67		Anaerolineales		bacterium		UBA5797		DIDP00000000	
68		Chloroflexi		bacterium		UBA4669		DHLL00000000	
69		Chloroflexi		bacterium		UBA4730		DHJC00000000	
70		Chloroflexi		bacterium		UBA4733		DHIZ00000000	
71		Chloroflexi		bacterium		UBA4735		DHIX00000000	
72		Chloroflexi		bacterium		UBA4736		DHIW00000000	
73		Chloroflexi		bacterium		UBA5189		DHWF00000000	
74		Chloroflexi		bacterium		UBA6019		DIZV00000000	
75		Dehalococcoides		mccartyi		UBA5818		DICU00000000	
76		Dehalococcoides		mccartyi		UBA5554		DIMY00000000	
77		Dehalococcoides		mccartyi		UBA5545		DINH00000000	
78		Dehalococcoides		mccartyi		UBA5853		DJGF00000000	
79		Dehalococcoides		mccartyi		UBA5846		DJGM00000000	
80		Dehalococcoidia		bacterium		UBA3088		DFBE00000000	
81		Dehalococcoidia		bacterium		UBA4086		DFYI00000000	
82		Dehalococcoidia		bacterium		UBA4087		DFYH00000000	
83		Dehalococcoidia		bacterium		UBA4462		DGOQ00000000	
84		Dehalococcoidia		bacterium		UBA5760		DIFA00000000	
85		Leptolinea		sp		UBA4782		DHHC00000000	
86		Nitrospinaceae		bacterium		UBA3496		DFQF00000000	

Abbreviation: MAG, metagenome‐assembled genome.

**Table A7:** Model data by domain/gene tree

Gene/ORF	Figure no.	Substitution model (selected by IQ Tree MF)
C1, no outgroup	A8	WAG+F+R4
C1, with outgroup	5	WAG+F+R6
C2	4, A2	LG+R7
NirS	4, A3	LG+F+R7
eNOR	6	LG+F+R6

Abbreviation: ORF, open reading frame.
